# Ultrathin Two-Dimensional Fe–Co Bimetallic Oxide Nanosheets for Separator Modification of Lithium–Sulfur Batteries

**DOI:** 10.3390/molecules27227762

**Published:** 2022-11-11

**Authors:** Jun Pu, Yun Tan, Tao Wang, Xiaomei Zhu, Shanshan Fan

**Affiliations:** 1 Key Laboratory of Functional Molecular Solids, Ministry of Education, College of Chemistry and Materials Science, Anhui Normal University, Wuhu 241002, China; 2 Anhui Provincial Engineering Laboratory for New-Energy Vehicle Battery Energy-Storage Materials, Anhui Normal University, Wuhu 241002, China

**Keywords:** two-dimensional materials, multifunctional separators, CoFe_2_O_4_, lithium–sulfur batteries

## Abstract

The shuttle effect is understood to be the most significant issue that needs to be solved to improve the performance of lithium–sulfur batteries. In this study, ultrathin two-dimensional Fe–Co bimetallic oxide nanosheets were prepared using graphene as a template, which could rapidly catalyze the conversion of polysulfides and inhibit the shuttle effect. Additionally, such ultrathin nanostructures based on graphene provided sufficient active sites and fast diffusion pathways for lithium ions. Taking into account the aforementioned benefits, the ultrathin two-dimensional Fe–Co bimetallic oxide nanosheets modified separator assembled lithium–sulfur batteries delivered an incredible capacity of 1044.2 mAh g^−1^ at 1 C and retained an excellent reversible capacity of 859.4 mAh g^−1^ after 100 cycles. Even under high loading, it still achieved high area capacity and good cycle stability (92.6% capacity retention).

## 1. Introduction

Cost-effective lithium-ion batteries were once the linchpin of rechargeable batteries for the electronics, electric vehicles and other energy industries. However, conventional batteries based on graphite and lithium cobalt dioxide are no longer able to meet the growing energy demand [1]. The lithium–sulfur batteries with ultrahigh theoretical capacity have been created for the limitation of poor energy density of these conventional batteries [2]. Theoretically, lithium–sulfur batteries based on Li + S → Li_2_S redox reaction can obtain a high capacity of 1675 mAh g^−1^ and an amazing energy density of 2600 Wh kg^−1^ at stable discharge voltage [3]. Unfortunately, the shuttle effect caused by soluble polysulfides (Li_2_S_n_, 4 ≤ *n* ≤ 8) diffusion between the anode and cathode is a major factor in why high-performance lithium–sulfur batteries have not completely supplanted lithium-ion batteries as the market leader. This shuttle effect is a result of the particular charging and discharging mechanism of lithium–sulfur batteries. Chain polysulfides accumulate on the surface of the separator or electrode and mutate with fresh polysulfides, finally forming some insoluble substances (mainly Li_2_S/Li_2_S_2_). This not only consumes the active material and reduces the capacity of the cell, but also blocks the active site and ion channel, resulting in slow reaction kinetics [4,5]. Therefore, how to suppress the shuttle effect is very important.

One of the most widely utilized strategies to address the aforementioned shuttle effect is to modify the design of functionalized separators [6,7]. For modified materials, carbon, polymers and inorganic substances are the most extensive choices [8,9,10,11]. Among them, carbon-based materials, such as activated carbon, carbon nanotubes and graphene, can speed up the charge transfer due to strong electrical conductivity. However, because of the weak interaction between carbon substrates and polysulfides, they perform slightly less effectively in the adsorption and capture of polysulfides [9,10,11], while polymers involve complicated preparation processes [8]. Transition metal oxides, due to the special electronic structure, can easily form “polar” interfaces and generate active sites that can adsorb or trap polysulfides, preventing the latter from moving between the anode and cathode [12,13,14,15]. For instance, Zhang et al. have described the use of CeO_2_ in the design of a modified separator to suppress the shuttle effect [16].

As special metal oxides, bimetallic oxides often have strong electrical conductivity and electrochemical activity [17,18]. They are not only proved to have good polysulfide adsorption function, but also can play a catalytic effect on polysulfide conversion [19]. However, for previous reports, most of the materials are bulk, meaning they are tens of nanometers or even micrometers thick [20,21]. In fact, for polysulfides, the adsorption and catalysis only act on the surface molecules of the catalyst, and the thick block is not conducive to the improvement of the energy density of the electrode [22]. Therefore, the thinning of the separator modification material is an important symbol for the practical application of batteries. It can not only maximize the utilization rate of the materials, but more importantly, small particles will expose more abundant active sites and improve the reaction kinetics [23,24].

Herein, this work developed ultrathin two-dimensional Fe–Co bimetallic oxide (CoFe_2_O_4_) nanosheets for use as a modified separator in lithium–sulfur batteries, taking into account the properties of the aforementioned materials. CoFe_2_O_4_ showed a two-dimensional structure that prevented particle aggregation during cycling. At the same time, the ultrathin structure lamellar layer made it easier to diffuse the charge, and wide lamellae provided plenty of Fe and Co active sites to completely interact with polysulfides [25,26,27,28,29]. In comparison to conventional Celgard-2400 separator, the results showed that the lithium–sulfur battery modified separator with CoFe_2_O_4_ nanosheets had improved electrochemical performance. It exhibited an ultra-high capacity of 1259.9 mAh g^−1^ at 0.2 C and 672.1 mAh g ^−1^ and 2 C. In the course of 500 cycles, the capacity decay rate of each cycle was only 0.1%. Even at a high load of 3.2 mg cm^−2^, it could still be as high as 2.94 mAh cm^−2^ and maintained good stability.

## 2. Results and Discussion

**Figure 1:**
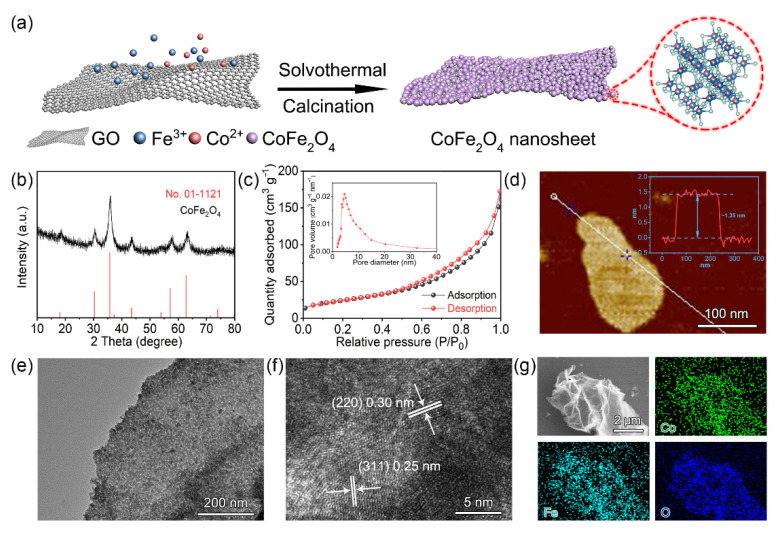
(**a**) Schematic illustration of the preparation processes of CoFe_2_O_4_ nanosheets. Phase characterization of the prepared CoFe_2_O_4_: (**b**) XRD; (**c**) N_2_ adsorption–desorption isotherm, inset: pore size distribution; (**d**) AFM image and the corresponding height profiles (inset); (**e**) TEM image; (**f**) HRTEM image; (**g**) areal elemental mapping images.

Figure 1a shows the fabrication process of the ultrathin CoFe_2_O_4_ nanosheets. Using graphene as a template, the Fe–Co precursors reacted with the oxygen-containing functional groups to form a sheet layer on the graphene surface. As-obtained dried precursors were calcined at 400 °C for 2 h in an atmosphere of air. The metal oxide was then directed to grow laterally in the two-dimensional plane to form a nanosheet shape during the subsequent calcination process [30,31,32]. The X-ray powder diffractometer (XRD) data of the substance created by the aforementioned procedure is shown in Figure 1b, and all diffraction peaks could exactly match the distinctive peaks of CoFe_2_O_4_ (JCPDS No. 01-1121). The crystal planes (111), (220), (311), (400), (511) and (440) were represented by the diffraction peaks that were positioned at 18.1°, 30.3°, 35.7°, 43.5°, 57.2° and 62.7°, respectively. The near-absence of graphene features meant that most of the graphene had been removed during high-temperature heat treatment. The very small amount of leftover graphene would somehow boost the conductivity of CoFe_2_O_4_. The substance was further examined using N_2_ adsorption–desorption resolution at 77 K, and the result in Figure 1c demonstrated that the two-dimensional CoFe_2_O_4_ nanosheets presented a significant Brunauer–Emmett –Teller (BET) surface area of 89.75 m^2^ g^−1^. This suggested that it possessed the high surface area of two-dimensional materials, which was advantageous for producing a lot of active sites for interacting with polysulfides. In addition, the inset reveals that the pore size distribution was around 5 nm, which would provide lithium ions quick transit pathways.

The morphology of the object under transmission electron microscopy (TEM) is shown in Figure 1e. Obviously, a two-dimensional CoFe_2_O_4_ nanosheet was made up of many ultra-fine nanoparticles with transverse sizes of about 7 nm. According to previous Reports, the oxygen-containing functional groups on the graphene surface initially coupled with Fe^3+^ and Co^2+^ and then directed the metal oxides to self-assemble in the two-dimensional plane using graphene as a template, generating this ultrathin two-dimensional structure [30]. Figure 1f shows the high-resolution transmission electron microscopy (HRTEM) image. The CoFe_2_O_4_ crystal presented a lattice spacing of 0.30 nm for the (220) crystal plane and 0.25 nm for the (311) crystal plane. From the energy dispersive spectroscopy (EDS) mapping and scanning electron microscopy (SEM) results in Figure 1g and Figure S1, it can be seen that the material revealed thin morphology and uniform element distribution. The thicknesses of the nanosheets further measured by atomic force microscopy (AFM) were only around 1.35 nm (Figure 1d), which was consistent with TEM.

**Figure 2:**
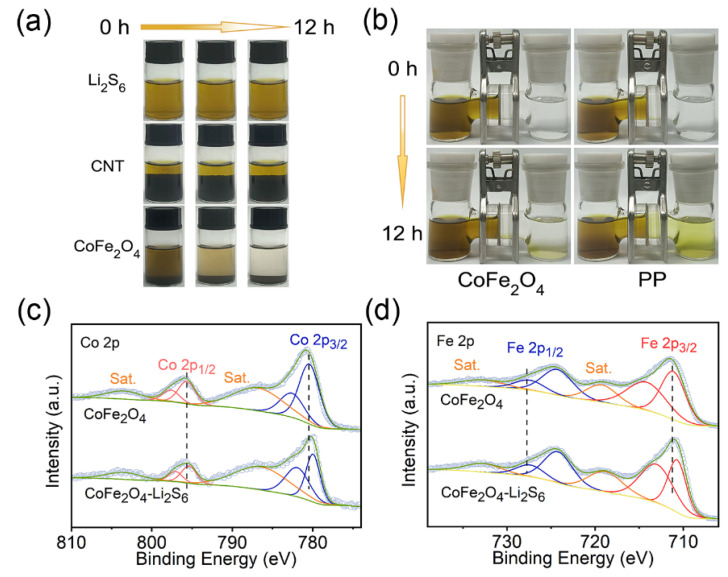
(**a**) Visual adsorption experiment; (**b**) lithium polysulfide penetration test; (**c**) Co 2p; and (**d**) Fe 2p XPS spectra of CoFe_2_O_4_ sample before and after Li_2_S_6_ adsorption. The curve of the grey circle connection is the data point of the original data, and the curve fitted by the XPS analysis software is green. The yellow line is the baseline. For Figure (**c**): the orange curve represents the peak of the metal cobalt salt, and the red and blue curves represent the peak position of the Co 2P orbital. For Figure (**d**): the orange curve represents the peak of the metallic iron salt, and the red and blue curves represent the peak position of the 2P orbit of Fe.

To comprehend the adsorption capability of CoFe_2_O_4_ on polysulfides more visibly, visual adsorption tests are crucial. As seen in Figure 2a, the original Li_2_S_6_ solution was yellowish brown without adding any material. The same volume and concentration of Li_2_S_6_ solution were then added with equal amounts of carbon nanotubes and CoFe_2_O_4_, respectively, and the results were monitored. Obviously, the solution containing carbon nanotubes still remained yellowish brown after 12 h, as can be seen with the naked eye, demonstrating the limited adsorption capacity of carbon nanotubes on polysulfides. According to previous studies, pure carbon has only weak adsorption between van der Waals forces for polysulfides [33,34]. After adding CoFe_2_O_4_, the hue of the solution brightened after 6 h and became transparent after 12 h, demonstrating its potent ability to anchor soluble polysulfides [35,36]. A lithium polysulphide permeation experiment was further conducted (Figure 2b), with pure electrolyte ether on one side of an H-shaped bottle and a specific concentration of Li_2_S_6_ on the other. A significant amount of the yellow Li_2_S_6_ infiltrated the other side with the middle barrier layer of pure Celgard (PP separator), indicating that the initial separator had no positive effect on the diffusion of polysulfides. In contrast, the color of the opposite solution was nearly unaltered in the system with the CoFe_2_O_4_-modified separator as the intermediate layer. Therefore, the latter was suitable for inhibition of the shuttle effect.

The outcomes of X-ray photoelectron spectroscopy (XPS) experiments on CoFe_2_O_4_ both before and after adsorbing Li_2_S_6_ are depicted in Figure 2c,d. The XPS plot of Co 2p demonstrated that the overall curve swung toward the low binding energy after polysulfide adsorption, which might be caused by the creation of CoS_X_ after the interaction of Co atoms with Li_2_S_6_. Due to Co acquiring electrons, the valence of Co element in CoS_X_ was lower than that in the +3-valence state of CoFe_2_O_4_ [37,38,39,40]. For the negative shift of the XPS curve for Fe 2p orbital, the same cause was investigated. After adsorption of Li_2_S_6_, the intensity of the two peaks at ~722.7 eV at 2p_1/2_ and ~709.2 eV at 2p_1/2_ increased slightly, similar to previous reports, possibly due to the formation of a small amount of Fe–S [41,42].

**Figure 3:**
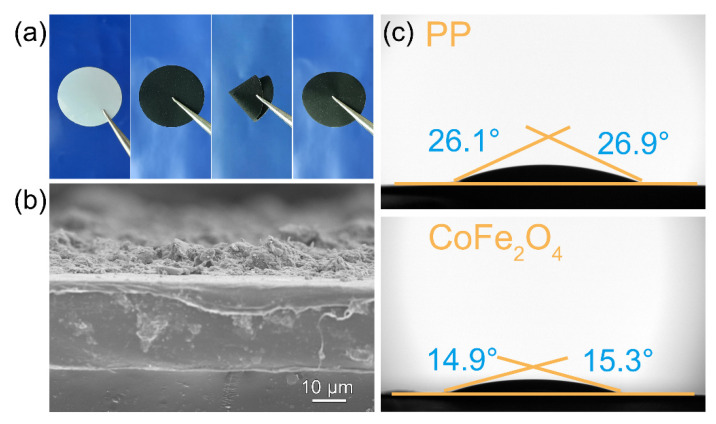
(**a**) Optical photographs of the folding and unfolding experiments of CoFe_2_O_4_-Celgard and pure Celgard separators; (**b**) cross-sectional SEM of CoFe_2_O_4_ improved separator; (**c**) the contact angle with electrolyte of different separators.

Optical images of the separator before and after CoFe_2_O_4_ modification are displayed in Figure 3a, where the material is uniformly covered on the surface of the commercial Celgard separator. After folding and re-expanding the modified separator multiple times, the coating on the surface remained intact, demonstrating favorable mechanical stability. The thinnest coating thickness could be achieved without increasing the cell volume to fully increase the diffusion rate of lithium ions. As shown in Figure 3b, the cross-sectional SEM image reveals that the CoFe_2_O_4_ coating on the separator was roughly 10 μm thick, lower than many previous reports (Table S1), fully meeting the coating-thickness criteria of the battery [43,44,45,46,47,48,49,50]. Furthermore, the electrolyte and coating should have a high degree of affinity for electrolyte penetration and ion diffusion. The coating materials with low levels of affinity are ineffective. Therefore, the contact angle test with the lithium–sulfur electrolyte was performed (Figure 3c). The CoFe_2_O_4_-modified separator outperformed the commercial separator in terms of contact angle. The contact angle size of the former was around 26°, whilst the latter was only about 15°. This indicated that the prepared coating not only did not hinder, but rather accelerated the diffusion of the electrolyte, which would be beneficial to the cell dynamics.

**Figure 4:**
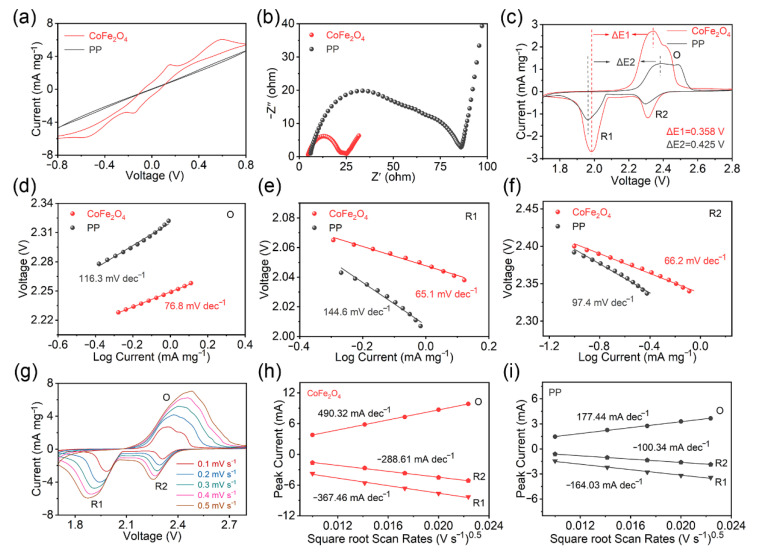
(**a**) CV and (**b**) EIS of symmetric cells with Li_2_S_6_ additive; (**c**) CV curves of sulfur cathode with different separators; (**d**–**f**) the corresponding Tafel plots and fitted slope values of oxidation and reduction peaks; (**g**) CV curves with CoFe_2_O_4_-modified separator at various scan rates; (**h**,**i**) plots of the CV peak current versus the square root of the scan rates of lithium–sulfur batteries based on pure and CoFe_2_O_4_-modified separators. For Figure (**h**): the red circle represents the data points corresponding to the oxidation peak (O) in Figure (**g**), and the triangle and pentagon correspond to the data points of the first reduction peak (R1) and the second reduction peak (R2), respectively. For Figure (**i**): Figure (**i**) is a graph between the CV peak current of the lithium-sulfur battery based on the PP separator and the square root of the scan rate, so the upper left corner is marked with PP. The gray circle represents the data points corresponding to the oxidation peak (O) in Figure S4, and the triangle and pentagon correspond to the data points of the first reduction peak (R1) and the second reduction peak (R2), respectively.

In addition to the adsorption function, the coating is also important for the catalytic acceleration of the polysulfide conversion process. In order to further explore the effect of the CoFe_2_O_4_-modified layer on the reaction kinetics of lithium–sulfur batteries, a series of electrochemical behaviors were carried out. The cyclic voltammetry (CV) curves (0.5 mV s^−1^) of the pure separator and the CoFe_2_O_4_-modified separator were measured in symmetric cells. As shown in Figure 4a, the CV curve of the original separator almost did not have any peak, while the obvious redox peaks and higher current response appeared in the CoFe_2_O_4_-modified system, indicating that the latter provided a strong catalytic activity for polysulfides [10]. To more clearly demonstrate the superior catalytic and lithium-ion diffusion capabilities of the coating, a pure separator and a CoFe_2_O_4_-modified separator were assembled into lithium–sulfur batteries (with ~68% sulfur content in CNT@S cathode, Figure S2). The electrochemical impedance spectra (EIS) of the two separators and the equivalent circuit used to fit the obtained EIS spectra are displayed in Figure 4b and Figure S3. The diffusion resistance decreases with the increasing slope of the diagonal line in the low-frequency area, and *R_ct_* (charge transfer resistance) increases with increasing semicircle size in the medium-frequency zone [51]. As can be observed in the figure, the impedance profile of the CoFe_2_O_4_-modified separator was significantly lower than the other one in terms of diffusion resistance and *R_ct_*.

At a sweep rate of 0.1 mV s^−1^, the CV tests were then conducted (Figure 4c). Both cells showed two distinct reduction peaks, representing the conversion of S_8_ to soluble polysulfides (R_2_) and the subsequent formation of solid Li_2_S_2_/Li_2_S (R_1_), respectively. The broad oxidation peak (O) corresponded to the opposite process [52]. It was obvious that the changed separator had a lower peak spacing (Δ*E*) and a higher current density than the unmodified one. The cell based on the CoFe_2_O_4_-modified separator exhibited an O-peak and R_1_-peak spacing of 0.358 V, whereas the cell assembled with the standard PP separator had a peak spacing of 0.425 V. The obvious polarization of the latter indicated that the reaction kinetics were slow. Additionally, to derive the matching Tafel slopes, the current densities of the anode and cathode peaks in Figure 4c were independently fitted to the overpotential. As shown in Figure 4d–f, the corresponding results of the modified separator, respectively, were 65.1 (R_1_), 66.2 (R_2_), and 76.8 (O) mV dec^−1^, lower than those of the unmodified one (from 97.4 to 116.3 mV dec^−1^), which again indicated that CoFe_2_O_4_ has excellent polysulfide catalytic conversion activity [53]. To evaluate the advantage of the modified separator-assembled lithium–sulfur cell in terms of lithium-ion diffusion rate, CV curves at various sweep rates were performed in Figure 4g and Figure S4. Linear fit was achieved using the square root of the sweep rate and the redox peak current (Figure 4h,i). The lithium-ion diffusion coefficient (D) is positively correlated with line slope (I_p_/ν^0.5^) according to the Randles–Sevcik equation: I_p_ = (2.69 × 10^5^) n^1.5^SD^0.5^ − Cν^0.5^, where I_p_ is the peak current, *n* is the number of electrons transferred, S is the active electrode area, C is the lithium-ion concentration, and ν is the sweep rate [54]. Clearly, the line slopes of the cell assembled by the modified separator were significantly higher than those of the ordinary cell with pure Celgard separator, indicating that the CoFe_2_O_4_ coating promoted the diffusion of lithium ions. From the above electrochemical experimental results, the CoFe_2_O_4_-modified separator may have greatly enhanced the reaction kinetics of lithium–sulfur batteries from the standpoint of accelerating polysulfide conversion and ion diffusion rate.

**Figure 5:**
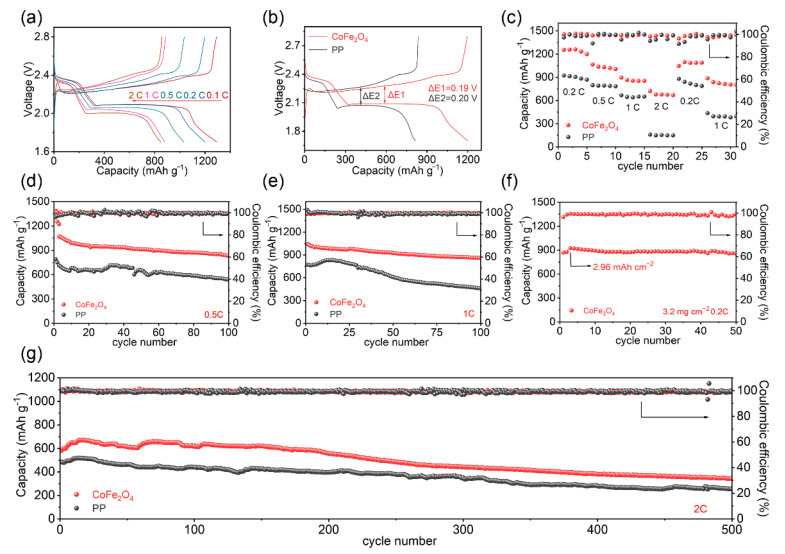
(**a**) Galvanostatic discharge–charge profiles of lithium–sulfur batteries with a CoFe_2_O_4_-modified separator at different rates; (**b**) comparison of discharge–charge curves at 0.2 C; (**c**) rate performance; (**d**,**e**,**g**) cyclic stability tests at 0.5, 1, and 2 C; (**f**) high load performance of the CoFe_2_O_4_-based battery. For Figure (**g**): 2C in the lower right corner means that both the CoFe_2_O_4_-modified separator and the PP separator assembled battery are tested for cycle stability at 2C. The two beating data points in the upper right corner are normal phenomena that occur during testing. The lithium-sulfur battery assembled without a modified separator has undergone hundreds of charge and discharge processes inside the battery after a long cycle. The consequences of the shuttle effect in this process will lead to unstable charging and discharging, so the data points corresponding to the Coulombic effect fluctuate.

Figure 5a reveals the discharge–charge profiles at various rates (0.1–2 C). The cell with the CoFe_2_O_4_-modified separator exhibited steady charge–discharge plateaus, which highlighted the robust redox reaction kinetics. Consistent with the results of earlier CV curves (Figure 4c), the polarization voltage of the CoFe_2_O_4_-based electrode (0.19 V) was lower than that of the pure Celgard separator (Figure 5b). The rate performance of the two cells was captured and displayed in Figure 5c. It was clear that the battery with the Fe–Co bimetallic oxide nanosheets modified separator obtained the better reversible capacities of 1259.9, 1064.8, 895.7 and 720.7 mAh g^−1^ at 0.2, 0.5, 1 and 2 C, much higher than those of the ordinary one. Especially at the current density of 2 C, with the assistance of CoFe_2_O_4_, the capacity maintained the same degree of reduction, while the cell based on the pure Celgard separator rapidly decreased to only 158.2 mAh g^−1^. During the fast charge–discharge process, the CoFe_2_O_4_ based lithium–sulfur battery showed more effective lithium-ion diffusion and polysulfide conversion, which could reduce the influence of the polarization phenomenon and exert the capacity of the battery to some extent [27,36]. After returning to 0.2 C, the modified separator still achieved a high capacity of 1095.7 mAh g^−1^, while the common separator was only 879.8 mAh g^−1^, indicating that the former was more stable.

In order to evaluate the stability of the lithium–sulfur batteries, constant-current charge–discharge cycling tests were carried out at different rates before and after modification (activated for two cycles at 0.2 C). As shown in Figure 5d, the capacity of pure Celgard fluctuated greatly before 60 cycles at 0.5 C, which might be because the shuttle effect makes polysulfide unable to complete the charge–discharge behavior well. For the CoFe_2_O_4_-modified separator, its capacity was stable during the cycle, with an initial capacity of 1072.5 mAh g^−1^, which remained at 835.2 mAh g^−1^ after 100 cycles. Figure 5e shows the cycle comparison at 1 C. The battery based on CoFe_2_O_4_ nanosheets maintained excellent catalytic and stability performance, with an impressive initial capacity of 1044.2 mAh g^−1^ and a capacity retention rate of 82.3% after 100 cycles, while the contrast sample dropped rapidly from 832.5 mAh g^−1^ to 461 mAh g^−1^. In order to further verify the service life of the modified lithium–sulfur battery, 500 charge–discharge cycles were carried out at 2 C. From Figure 5g, the improved cell maintained high capacity and low capacity decay during the first 200 cycles of the long cycle. Even at the 500th cycle, it still obtained a high capacity of 343.3 mAh g^−1^, which was a significant advantage over the pure PP separator cell.

The loading of a cathode active substance in the above assembled lithium–sulfur batteries was approximately 1 mg cm^−2^. In fact, in order to obtain a practical capacity, the loading often needs to be higher. Figure 5f shows the cycling performance of a CoFe_2_O_4_-based cell with a high sulfur loading of 3.2 mg cm^−2^. A considerable initial capacity of 924.9 mAh g^−1^ (2.96 mAh cm^−2^) could be assigned to the device at 0.2 C. After 50 cyclings, the outstanding stability resulted in a consistent capacity of 856.7 mAh g^−1^. Taken together, with the help of the ultrathin Fe–Co bimetallic oxide nanosheets modified layer, the lithium–sulfur batteries exhibited considerable rate, stability and high loading properties.

## 3. Materials and Methods

### 3.1. Preparation of CoFe_2_O_4_ and Modified Separator

In the synthesis of ultrathin two-dimensional CoFe_2_O_4_ nanosheets, 120 mg of graphene oxide was first uniformly dispersed into 75 mL of ethylene glycol and sonicated to obtain solution A. Solution B was obtained by dispersing 1 mmol of Co (NO_3_)_2_▪6H_2_O and 2 mmol of Fe (NO_3_)_2_▪9H_2_O into 25 mL of ethylene glycol. The above two solutions were mixed together and stirred at room temperature for 1.5 h. Then, the resulting suspension was condensed and refluxed at 170 °C while stirring for 2 h. After natural cooling, the solids were separated by centrifugation, washed with deionized water and absolute ethanol, and vacuum-dried overnight at 80 °C. Finally, the dried powder was calcined in air at 400 °C for 2 h using a heating rate of 0.5 °C min^−1^ to obtain the final product.

For the CoFe_2_O_4_-modified separator. A slurry consisting of 70% CoFe_2_O_4_, 20% carbon nanotubes and 10% polyvinylidene fluoride (PVDF) binder was coated on one side of a commercial Celgard-2400 separator and dried under vacuum at 60 °C.

### 3.2. Materials Characterization

The morphology and microstructure were observed by SEM (Hitachi Regulus 8100, operated at 5 kV) and TEM (FEI Tecnai G^2^ 20). The elemental distribution was collected by EDS mapping through SEM at an accelerating voltage of 15 kV. The crystal structure and phases were determined by XRD (Rigaku Smart Lab) at a wavelength of 1.5418 Å with copper Kα radiation. The specific surface area and pore size distribution were analyzed at a particulate ASAP2460 analyzer with BET calculation. The wettability of the electrolyte was measured by a contact angle (Theta) test. Elemental analysis was performed using XPS (Thermo Scientific K-Alpha). Thickness of material was measured by AFM (Bruker Dimension Icon).

### 3.3. Visualization Lithium Polysulfide Adsorption

Briefly, Li_2_S and sulfur power (molar ratio, 1:5) were added to a mixed solution of 1,3-dioxolane (DOL) and 1,2-dimethoxymethane (DME) in the volume ratio of 1:1 at 60 °C with stirring to obtain a Li_2_S_6_ solution of 0.027 M. The same mass of CoFe_2_O_4_ nanosheets and carbon nanotubes were added to a certain amount of the above solution, respectively.

### 3.4. Battery Assembly and Electrochemical Measurements

The compound sulfur cathode was obtained by melting sublimated sulfur and carbon nanotubes with a mass ratio of 7:3 at 155 °C (Figure S2). A certain mass of as-obtained sulfur composite material (80 wt%), acetylene black (10 wt%), PVDF (10 wt%) and N-methyl-pyrrolidone (NMP) was taken and mixed thoroughly and coated on Al foil. The cathode with a loading of about 1–3.2 mg cm^−2^ was obtained by vacuum drying (1 mg cm^−2^ for normal tests and 3.2 mg cm^−2^ for high loading tests). Metallic lithium was used as the anode. The DOL/DME (*v*/*v*, 1:1) mixture of dissolved 0.5 M LiTFSI and LiNO_3_ was the electrolyte. A commercial Celgard-2400 or CoFe_2_O_4_ modified Celgard-2400 was the separator, and the above parts were assembled as a coin cell (CR-2032).

For symmetric cell, carbon nanotubes or CoFe_2_O_4_ mixed with acetylene black, PVDF binder, and NMP were coated on Al foil and dried as electrode; 0.5 M Li_2_S_6_ (DOL/DME mixed LiTFSI solution) as the electrolyte, commercial Celgard-2400 as the separator and the above parts were assembled into a coin cell.

Constant-current charge–discharge tests were performed on a LAND system (CT-2001) with a voltage range of 1.7–2.8 V. The CV and EIS (10 mHz–100 kHz) were tested on an electrochemical workstation (CHI 660E).

## 4. Conclusions

In summary, ultrathin two-dimensional CoFe_2_O_4_ nanosheets were synthesized using graphene as template by a simple two-step method. The superb ability of this Fe–Co bimetallic oxide adsorption to capture polysulfides and the ultrathin two-dimensional lamellar structure provided abundant active sites for the redox reaction and fast diffusion channels for lithium ions. These variables promoted the polysulfide catalytic conversion and lithium-ion diffusion, giving lithium–sulfur batteries a high initial capacity and a relatively slower capacity degradation rate. As a result, the lithium–sulfur battery with the CoFe_2_O_4_-modified separator showed an amazing capacity of 1259.9 mAh g^–1^ at 0.2 C and a high 82.3% capacity retention rate after 100 cycles at 1 C. The bimetallic oxide nanosheets created and manufactured in this study provided a new approach to successfully circumvent the low utilization of metal oxides and increased the potential applications of two-dimensional materials in the field of lithium–sulfur batteries.

## Data Availability

Not applicable.

## Supplementary Materials

The following supporting information can be downloaded at: https://www.mdpi.com/article/10.3390/molecules27227762/s1,

## Sample Availability

Samples of the compounds are available from the authors.
