# Supplementary material for: Ultrathin Two-Dimensional Fe–Co Bimetallic Oxide Nanosheets for Separator Modification of Lithium–Sulfur Batteries
Source: Molecules. 2022 Nov 11;27(22):7762. doi: 10.3390/molecules27227762 (PMC9693558; doi:10.3390/molecules27227762)
Supplement: Supplementary file 1 [file molecules-27-07762-s001.zip › molecules-2023311-supplementary.pdf]

Supporting Information

Ultrathin two-dimensional Fe-Co bimetallic oxide nanosheets for separator modification of lithium-sulfur batteries

Jun Pu, Yun Tan, Tao Wang, Xiaomei Zhu, Shanshan Fan*

Correspondence: jpu@ahnu.edu.cn (J.P.)

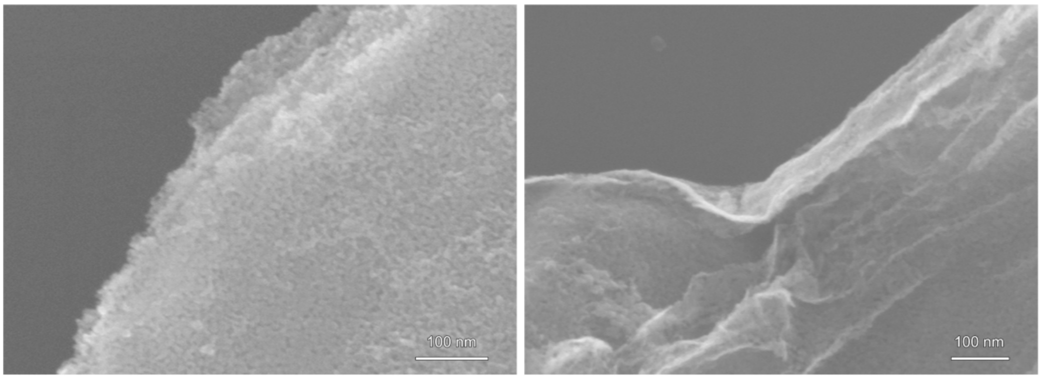

Figure S1. SEM of $CoFe_2O_4$ nanosheets.

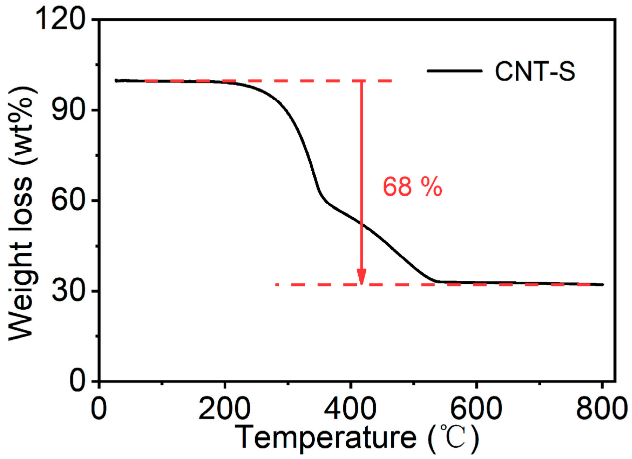

Figure S2. TGA curve of the CNT-S composite.

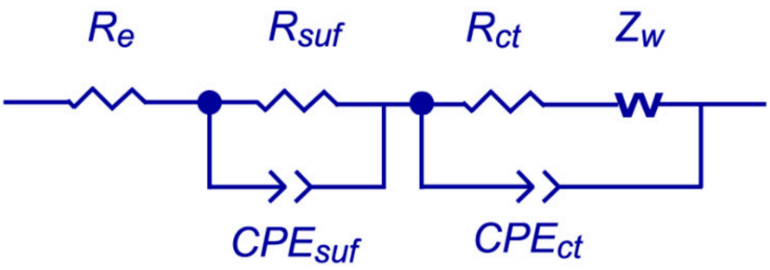

Figure S3. The equivalent circuit used to fit the obtained EIS spectra [1].

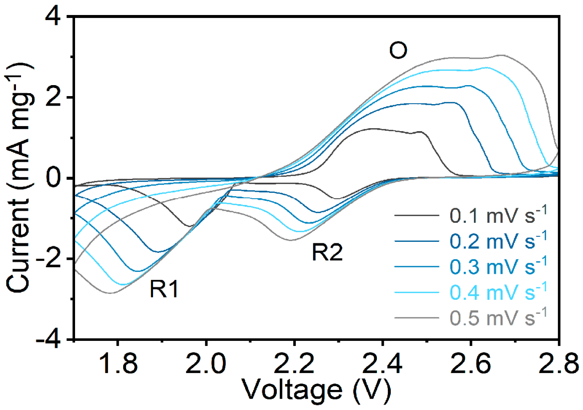

Figure S4. CV curves at various scan rates based on PP separator batteries.

Table S1. Comparison of this $CoFe_2O_4$ coating layer compared with other reports.

Materials	Thickness (μm)	Rate performance ($mAh \cdot g^{-1}$)	Ref.
EVAs	18	507.3 (0.2 C)	[2]
MWCNTs/CeO_2	10.5	898.3 (0.2 C)	[3]
Ni/NiO-C	12	1333 (0.2 C)	[4]
Nb_2O_5/CNT	7.5	998 (0.2 C)	[5]
HGSCS	50	1172.3 (0.2 C)	[6]
**$CoFe_2O_4$**	**10**	**1259.9 (0.2 C)**	**This work**

References

1. Pu, J.; Wu, J.; Tan, Y.; Yu, S., Cobalt nitride nanocrystals coated separator as multifunctional interlayer for improving polysulfides regulation. *J. Alloy. Compound.* **2022,** *920*, 165964.
2. Wei, Z.; Zhang, N.; Feng, T.; Wu, F.; Zhao, T.; Chen, R., A copolymer microspheres-coated separator to enhance thermal stability of lithium-sulfur batteries. *Chem. Eng. J.* **2022,** *430*, 132678.
3. Zhu, W.; Zhang, Z.; Wei, J.; Jing, Y.; Guo, W.; Xie, Z.; Qu, D.; Liu, D.; Tang, H.; Li, J., A synergistic modification of polypropylene separator toward stable lithium-sulfur battery. *J. Membrane Sci.* **2020,** *597*, 117646.

4. Pu, J.; Wang, T.; Zhu, X.; Tan, Y.; Gao, L.; Chen, J.; Huang, J.; Wang, Z., Multifunctional Ni/NiO heterostructure nanoparticles doped carbon nanorods modified separator for enhancing Li-S battery performance. *Electrochim. Acta* **2022,** *435*, 141396.
5. Zhan, L.; Zhou, X.; Luo, J.; Fan, X.; Ning, X., Urchin-like Nb_2O_5/CNT modified separator for lithium-sulfur batteries. *Int. J. Hydrogen Energy.* **2022,** *47*, 27671–27679.
6. Li, P.; Deng, J.; Li, J.; Wang, L.; Guo, J., Hollow graphene spheres coated separator as an efficient trap for soluble polysulfides in Li-S battery. *Ceram. Int.* **2019,** *45*, 13219–13224.
